# An unusual exacerbation of chronic obstructive pulmonary disease (COPD) with herpes simplex tracheitis: case report

**DOI:** 10.1186/1752-1947-1-91

**Published:** 2007-09-19

**Authors:** Alison C Boland, Elizabeth H Iveson, Mark W Elliott

**Affiliations:** 1 Department of respiratory medicine, St James's university hospital, Leeds, UK

## Abstract

Chronic obstructive pulmonary disease (COPD) is a common cause of morbidity in the UK and is increasingly seen in elderly patients, often requiring multiple courses of steroids. We present a case of a 72 year old lady with repeated exacerbations of COPD which did not respond to conventional treatment. Herpes simplex virus (HSV1) tracheobronchitis was diagnosed following a rigid bronchoscopy and her symptoms improved with intravenous acyclovir. This is the first published case of HSV tracheitis in a non immunosuppressed individual with chronic lung disease.

## Background

Herpes simplex virus (HSV1) infection may be considered in the differential diagnosis of patients with chronic lung disease not responding to conventional treatment. This infection is a rare, but potentially treatable, cause of exacerbations in such patients. Appropriate diagnostic studies should be performed to confirm the diagnosis and initiate therapy accordingly. Studies have documented a significant mortality related to herpes infection and a raised awareness of this condition is important to improve outcome in these patients [1,2].

We present a case of a 72 year old lady with repeated exacerbations of COPD which did not respond to conventional treatment.

## Case Presentation

A 72 year old lady, with known chronic obstructive pulmonary disease (COPD), was seen in the outpatient department with a six month history of progressive shortness of breath. Over this time she had suffered four exacerbations, requiring steroids and antibiotics, but no hospital admission. Previously her symptoms had been controlled with inhaled steroids, bronchodilators and as required home nebulisers. She also reported several episodes of streaky haemoptysis but there was no history of weight loss.

Two years previously she had suffered a myocardial infarction resulting in mildly impaired left ventricular function; there was no history of any HSV infection. She was an ex-smoker with a 50 pack year history. Medication included Tiotropium 18 micrograms od, Seretide 250 ii bd and Brycanyl inhalers, as required Salbutamol nebulisers, Monteleukast 10 mg od, Valsartan 80 mg od, Clopidogrel 75 mg od, Fluoxetine 20 mg od, Prednisolone 10 mg od and Ezetimibe 5 mg od.

Examination revealed widespread expiratory wheezing, there was no evidence of oral HSV; the remaining examination was unremarkable. Chest radiograph and baseline blood tests were all normal; her spirometry had remained stable over the last year FEV_1 _0.85 l/min, FVC 1.15 l/min.

Fibre-optic bronchoscopy was performed because of the haemoptysis. This showed widespread inflammation of the endobronchial tree with nodules throughout the mucosa of the trachea and both main bronchi. Bronchial washings, brushings and biopsies, showed active chronic inflammation with no malignant cells identified.

Subsequently, due to an acute deterioration in symptoms, she was admitted with an infective exacerbation of COPD. Despite treatment, her symptoms continued to deteriorate and she developed an inspiratory stridor. High resolution computerised tomography of her thorax showed a narrowing in the left main bronchus, with no lymphadenopathy. A repeat bronchoscopy was unchanged revealing widespread mucosal abnormality, with a nodular appearance. Copious mucus plugging was seen and cultures of the secretions isolated pseudomonas aeruginosa. She was commenced on intravenous Ceftazadime with little improvement.

Further investigations, including immunoglobulins, complement, specific antibody levels and a vasculitis screen were all normal. Repeat bronchoalveolar lavage (BAL) was inconclusive and viral cultures of BAL samples were negative.

A rigid bronchoscopy, performed to obtain a larger biopsy sample, revealed partial stenosis and irregularity of the main bronchi. Histological examination showed foci of ulceration with multi-nuclear cells and grand blast intra-nuclear viral inclusions peripherally, suggestive of herpetic infection. Immunohistochemistry confirmed the presence of herpes simplex and PCR for HSV1 was also positive.

She was reviewed by the immunologist who found no immune deficiency. After two weeks on intravenous acyclovir (5 mg per kg tds), her symptoms improved and she was discharged home.

Following discharge, repeat bronchoscopies have shown significant improvement of the abnormal mucosa and nodularity. A CT bronchoscopy was also performed which demonstrated persistent narrowing of her left main bronchus. Subsequent to her treatment with intravenous acyclovir, she underwent two further admissions with episodes of dyspnoea and mild stridor. These responded to further courses of intravenous acyclovir and antibiotics. It was therefore decided to commence maintenance acyclovir 400 mg bd initially then 200 mg bd after six months. She has remained well on this and has only required one admission for an exacerbation in the following two years.

## Discussion

This is the first published case of herpes simplex tracheitis in the non-immunocompromised patient with chronic lung disease. It has however, been suggested that herpetic respiratory infections are commoner in patients with underlying lung disease [3]. HSV causes a latent infection resulting in a potential for recurrence particularly in the elderly or immunosuppressed. In this case, repeated courses of steroids for COPD exacerbations and low dose maintenance prednisolone, were thought to have made the patient more susceptible to viral infections however, formal immunological tests were normal.

Lower respiratory tract HSV infections have been reported in newborn infants, patients with burns, patients with Acquired Immunodeficiency Syndrome (AIDS) and those who have been intubated [1,2,4-6].

The virus source is usually from the oropharynx. Several patterns of pulmonary damage can occur, with tracheobronchitis the most common manifestation. Ulceration of the trachea may be associated with necrotizing pneumonia. The surface of the ulcerated area is covered with a fibrinopurulent exudate containing necrotic cells, nuclear debris, fibrin and inflammatory cells. The histological appearances are often attributed to a bacterial infection with viral infection not being suspected [4].

Isolation of the virus from respiratory secretions alone does not confirm the diagnosis, as 1–5% of the population excretes herpes virus in the oropharynx without symptoms [4]. Diagnosis is best made in combination with viral culture, PCR and the presence of characteristic features (intra nuclear inclusions) demonstrated on histology.

Patients with herpes infection of the respiratory tract may develop severe airway obstruction and present with stridor. This occurs due to necrosis of large amounts of epithelium resulting in a thick pseudo membrane. Tracheal dilation and sequential bronchoscopic excisions of granulation tissue are required to relieve the obstruction [4,5,7-9].

## Conclusion

Many patients in the UK are exposed to HSV and its role in difficult to treat exacerbations of COPD may be underestimated. Diagnosis may be considered in patients with chronic lung disease, especially during exacerbations of COPD who are not responding to conventional treatment. It should also be considered in elderly patients, those who are difficult to wean from ventilation and in the immunocompromised [6,9,10].

Appropriate diagnostic studies should be undertaken and documented isolation of HSV1 obtained before appropriate treatment is commenced. Studies have documented a significant mortality related to HSV infection and a raised awareness of this condition is important to improve the outcome in these patients [1,2].

## Competing interests

The author(s) declare that they have no competing interests.

## Authors' contributions

ACB: Case review, literature review and drafting the manuscript.

LHI: Literature review and editing manuscript.

MWE: Manuscript critique and review.

All authors have read and approved the final manuscript

## References

[B1] Nash G Necrotizing tracheobronchitis and bronchopneumonia consistent with herpetic infection Human Pathology 1972 3 283 91 4337636 10.1016/s0046-8177(72)80082-0

[B2] Herout V Vortel V Vondrackova A Herpes simplex involvement of the lower respiratory tract American Journal of Clinical Pathology 1966 46 411 19 5924008 10.1093/ajcp/46.4.411

[B3] Frable WJ Frable MA Senev FD Jr Virus infections of the respiratory tract; cryopathologic and clinical analysis Acra cytol (Baltimore) 1977 21 32 6 189546

[B4] Dail DH Hammar SP Pulmonary Pathology 1988 Publishing company: Sprinder-Verlag

[B5] McMarthy DW Qualman SJ Rudman DT Wiet GT Besner GE Herpetic tracheitis and brachial plexus neuropathy in a child with burns Journal of burn care & Rehabilitation 1999 20 5 377 81 10.1097/00004630-199909000-00009 10501325

[B6] Baras L Farber CM Van Cooren JP Parent D Herpes simplex virus in a patient with the acquired immunodeficiency syndrome European Respiratory Journal 1994 7 11 2091 3 7875288

[B7] St John RC Pacht ER Tracheal stenosis and failure to wean from mechanical ventilation due to herpetic tracheitis Chest 1990 98 6 1520 2 2245699 10.1378/chest.98.6.1520

[B8] Nadel S Offit PA Hodinka RL Gesser RM Bell LM Upper airway obstruction in association with perinatally acquired herpes simplex virus infection Journal of Paediatrics 1992 120 1 127 9 10.1016/S0022-3476(05)80616-X 1731009

[B9] Vitale VJ Saimen L Haddad J Jr Herpes laryngitis and tracheitis causing respiratory distress in a neonate Archives of Otolaryngology-head and neck surgery 1993 119 2 239 40 10.1001/archotol.1993.01880140129021 8381288

[B10] Prellner T Flamholc L Haidl S Lindholm K Widell A Herpes simplex virus; the most frequently isolated pathogen in the lungs of patients with severe respiratory distress Scandinavian Journal of Infectious Diseases 1992 24 3 283 92 1324521 10.3109/00365549209061333

